# Supplementary material for: Baseline Characterization of the Gut Microbiota of Field and Colony Populations of Phlebotomus tobbi and Preliminary Assessment of the Anti-Leishmanial Activity of Cultivable Bacteria
Source: Pathogens. 2026 Jun 23;15(7):658. doi: 10.3390/pathogens15070658 (PMC13416292; doi:10.3390/pathogens15070658)
Supplement: Supplementary file 1 [file pathogens-15-00658-s001.zip › pathogens-4357168-File S1/Taxa2ASV_Decomposer/Family/f__Enterobacteriaceae/seqs.mltree.pdf]

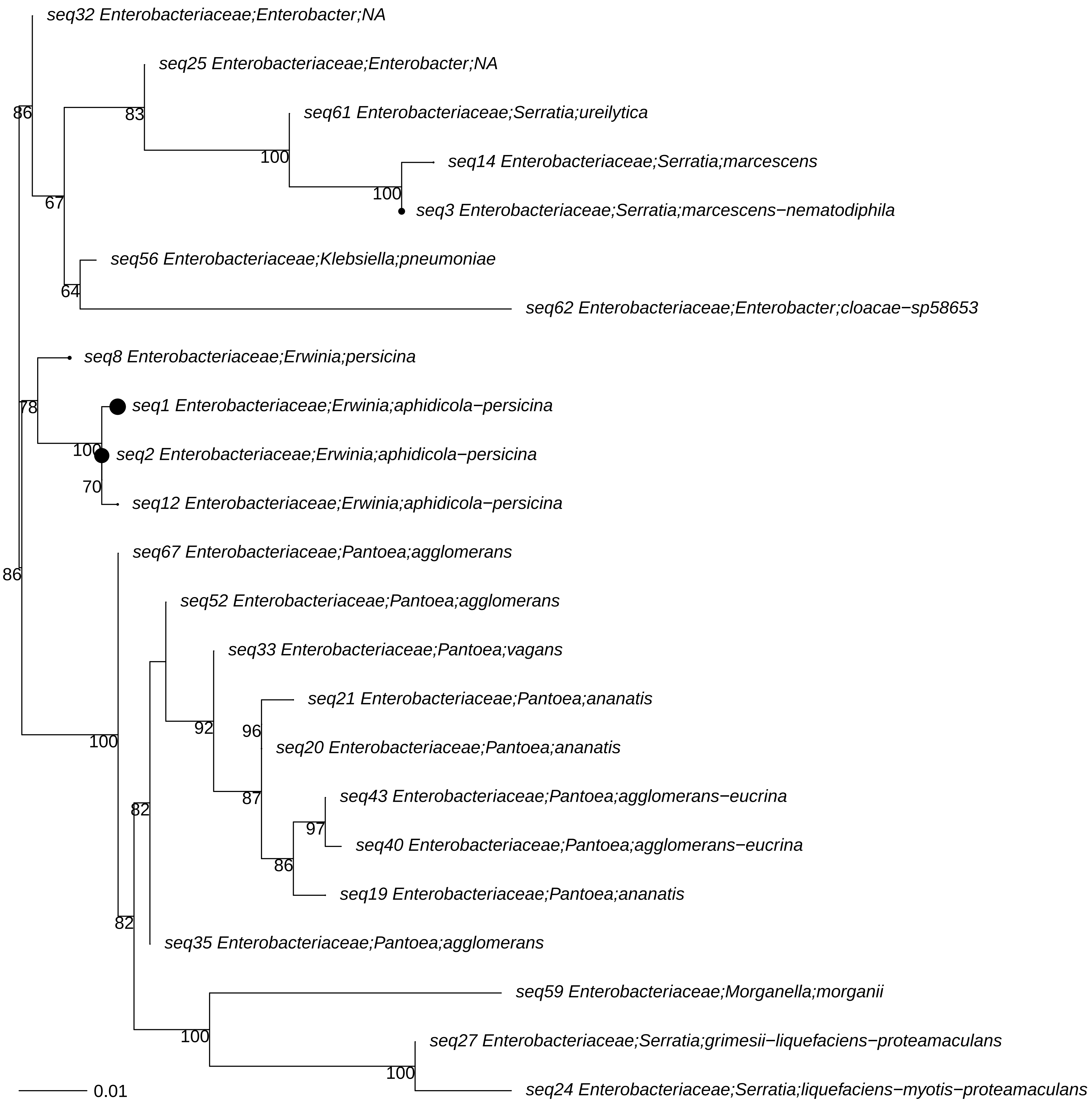
seq32 Enterobacteriaceae;Enterobacter;NA
seq25 Enterobacteriaceae;Enterobacter;NA
seq61 Enterobacteriaceae;Serratia;ureilytica
seq14 Enterobacteriaceae;Serratia;marcescens
seq3 Enterobacteriaceae;Serratia;marcescens−nematodiphila
seq56 Enterobacteriaceae;Klebsiella;pneumoniae
seq62 Enterobacteriaceae;Enterobacter;cloacae−sp58653
seq8 Enterobacteriaceae;Erwinia;persicina
seq1 Enterobacteriaceae;Erwinia;aphidicola−persicina
seq2 Enterobacteriaceae;Erwinia;aphidicola−persicina
seq12 Enterobacteriaceae;Erwinia;aphidicola−persicina
seq67 Enterobacteriaceae;Pantoea;agglomerans
seq52 Enterobacteriaceae;Pantoea;agglomerans
seq33 Enterobacteriaceae;Pantoea;vagans
seq21 Enterobacteriaceae;Pantoea;ananatis
seq20 Enterobacteriaceae;Pantoea;ananatis
seq43 Enterobacteriaceae;Pantoea;agglomerans−eucrina
seq40 Enterobacteriaceae;Pantoea;agglomerans−eucrina
seq19 Enterobacteriaceae;Pantoea;ananatis
seq35 Enterobacteriaceae;Pantoea;agglomerans
seq59 Enterobacteriaceae;Morganella;morganii
seq27 Enterobacteriaceae;Serratia;grimesii−liquefaciens−proteamaculans
seq24 Enterobacteriaceae;Serratia;liquefaciens−myotis−proteamaculans
86
83
100
100
67
64
78
100
70
86
92
96
100
87
97
82
86
82
100
100
0.01
